# American Dental Association

**Published:** 1896-10

**Authors:** 


Adjourned.

---

### AMERICAN DENTAL ASSOCIATION.

*August 4, 1896.—First Day.—Morning Session.*

The Thirty-sixth Annual Meeting of the American Dental Association was held at Saratoga Springs, N. Y., commencing August 4 1896.

The meeting was called to order at 11.25 by the president, Dr. J. Y. Crawford, and was opened by prayer by Dr. W. H. Morgan, of Nashville, Tenn.

Upon motion the call of the roll of membership and reading of the minutes of the last meeting were dispensed with.

The secretary announced the resignation of Drs. Gillette and Ames, which resignations were accepted. The secretary also read a communication from the Indiana State Association, stating that Dr. Cravens has been restored to membership in that society. A communication was read from Mrs. J. J. R. Patrick, widow of Dr. Patrick, enclosing bill for expenses incurred by Dr. Patrick in the examination of crania for this society.

*Dr. Peirce.*—As the Finance Committee of this society is quite familiar with the work of Dr. Patrick, I move that this matter be referred to them for action.

Motion carried.

Dr. Cushing read the report of the Publication Committee, in which attention is called to the delay in publishing the transactions, as the copy was in the hands of the publishers on October 28, 1895. The committee suggest that some arrangement be made with the publishers guaranteeing the publication of the transactions by the month of January following the meeting of the Association. The committee also presented a bill for expenses incurred.

Motion made that report be received and the suggestion therein approved.

*Dr. Crouse.*—As this is a question that has never arisen before, I think it would be wise for the Association to turn the matter over to the Executive Committee, to make definite arrangements. It may be that we are not in a position to dictate absolutely such terms as these. The question came up before the Executive Committee last night. It was suggested there that, when the sections are made up, if the corresponding secretary would then have the lists printed and sent to each member, that that would obviate a great drawback, which is that the sections do not know where their proper places are, and their reports do not come in properly. The Executive Committee should, of course, make the best terms they can with the publishers, but I do not know if they can dictate to them just when they are to be printed.

*Dr. Morgan.*—I think six months' time is plenty in which to print the transactions. I myself am connected with a publishing house, and we could get it out in six weeks; so I think we ought to get the transactions much earlier.

*Dr. Cushing.*—I want to say a word in regard to what Dr. Crouse said, and that is that the original proposition, which came from the publishing house that has done the work for many years, fixes the time itself, and it is very much shorter than they have taken to publish the transactions.

*Dr. Crouse.*—We give the parties the material as a compensation. If you insist upon the proceedings coming out all at once, you will not be able to make such good arrangements.

*Dr. Peirce.*—I am somewhat familiar with the action of the publishers. Those of you who have seen the copy already out must have noticed a great many very fine illustrations. They have been prepared at much expense and a great deal of time, and that may be offered as some apology for the time taken by the publishers.

*Dr. Boice.*—I would move that it be referred to the Executive Committee.

*Dr. Smith.*—It seems to me, before any definite action is taken, both sides should be heard. I have not heard any reason for the delay. If there are good reasons for it, we would like to know. If the transactions are published in the condition they are now, we cannot expect them as early as if we published them ourselves. If the transactions are necessarily delayed, the difficulty could be obviated by the manner suggested by Dr. Crouse,—that we have the organization of the sections earlier.

*Dr. Boice.*—About six weeks ago the transactions of the last meeting were published. They must have spent at least one thousand dollars in the publication, and if we had published them, it would have cost us at least fifteen hundred dollars.

Dr. Boice's motion seconded and carried.

The report of the corresponding secretary was read. In it Dr. Chase states that she has received no letters of importance during the past year. Letters of inquiry have been answered, and blank certificates for delegates to the meeting have been sent to all societies requesting them. Bill for postage and stationery accompanying this report, one dollar.

Report received.

REPORT OF THE TREASURER.

| | |
|---|---|
| Balance on hand | $606.93 |
| Collected at Asbury Park | 1244.90 |
| | $1851.83 |
| Paid out during the year | 572.55 |
| Balance | $1279.28 |

The expenditures were as follows:

| | |
|---|---|
| Secretary's salary for 1894–95 | $200.00 |
| Treasurer's salary | 100.00 |
| Reporter for session of 1895 | 125.00 |
| Expenses of corresponding secretary | 15.00 |
| Chairman of Section I. | 7.50 |
| Chairman of Section II. | 2.50 |
| Chairman of Section VII. | 15.60 |
| Expenses of chairman of Executive Committee | 32.50 |
| Printing Dr. Patrick's report | 30.00 |
| Chairman Publication Committee | 30.00 |
| Printing, stationery, postage, and expressage | 14.45 |
| | $572.55 |

Dr. Donnelly reported on behalf of the Committee on the Army Medical Museum. He stated that the museum contains more than thirty-five thousand specimens, and is open to the public. Its dental section may be made its most attractive department, and the greatest object lesson of its kind. A number of valuable and beautifully mounted specimens have recently been acquired, most of which are rare, many of which illustrate the effect of diseases of the maxillary bones and teeth. Many of the State and local societies have appointed auxiliary committees to act with this committee. It is impossible to detail the kind of specimens desired, but anything illustrative of any part of the subject of dentistry, which would throw light on the etiology or pathology of the teeth, jaws, etc., would be of great value. It is impossible to say in how many ways such a great depository as this museum reaches and teaches the thousands of intelligent and advanced students who come from everywhere, and return and impart the information acquired to others. The committee recommends,—

1. The appointment of five members of the Association as a National Committee, charged with the duty of building up a great national museum.

2. That the society recommend the appointment of auxiliary committees.

3. That one hundred dollars be appropriated and used with other donations in defraying the expenses of the National Committee.

The report was referred to the Executive Committee.

The president called for a report from the committee appointed to look after the question of introducing into the medical examination some inquiry as to the condition of the oral cavity in reference to the question of life insurance. The committee, not being ready to report, was passed for the present.

The president also called for reports from the Committee on the Priority of Invention of Dental Furnaces, and the Committee on the Union of the American Dental Association and the Southern Dental Association; both committees will report at a later session.

Dr. Fillebrown, however, stated, in reference to the committee as to the union of the two societies, that the matter has been considered, in connection with the Southern Dental Association, several times during the year, and the results are these: The Southern Dental Association appointed a committee last fall, at Atlanta, to confer with our committee, and last week they held a meeting at Asheville. They have decided to meet at Old Point Comfort on the first Tuesday of August, 1897, and they express the hope that it will be agreeable to have the American Dental Association meet there also at that time. I understand that the president and officers are empowered to consider some other place, if it be thought desirable. We have called a committee meeting for later in the day, and we will make another report.

### COMMITTEE ON STATE AND LOCAL SOCIETIES.

*Dr. Crouse.*—Dr. Kirk explained to me last night that he had not had time to attend to the work as he wished to. He asks to be relieved from that committee, and have some one put in his place who could attend to it. I would like to have the report postponed for another time, as I wish to bring it up in another form.

### COMMITTEE ON REVISION OF CONSTITUTION AND BY-LAWS.

*Dr. Fillebrown.*—The committee has had no meetings this year, and no correspondence. The time did not seem to have arrived for it. Later in the session probably there will be another report.

### REPORT OF THE EXECUTIVE COMMITTEE.

*Dr. Crouse.*—After Saratoga was selected last year as the place of meeting for this year, we appointed a local committee, and asked them to come here and investigate the place, and see where we could hold the meeting. The local committee decided on this hotel as furnishing the best accommodations for our meetings and meeting-room.

In regard to the programme, it will be remembered that at the last session this Association passed a resolution having in view more section work and less general meetings,—that is, having but one session a day, and having the work done in the sections. Several difficulties arose in that matter, growing out of the fact that the sections were not well organized this year. I wrote to the different chairmen to see what their preference was, and I found all the sections opposed to trying to adopt it this year, and the Executive Committee took it upon themselves to disobey that resolution, on the ground that we could not perfect the arrangements, and we therefore left these as they were last and previous years.

We offer this printed programme as the programme for the session. I might mention that Section I. wanted to carry out the resolution of last year as to section meetings, but the others did not.

Report adopted.

The second vice-president, Dr. Fillebrown, then took the chair while the president, Dr. J. Y. Crawford, read his address, an abstract of which follows:

Notwithstanding the great financial depression and much agitation in our country, considerable progress in our profession has obtained within the last twelve months, which it would be interesting and instructive at this time to relate and emphasize, but as there are other matters that more directly affect the future welfare of our vocation and the good of the whole people, I have concluded to call your attention to the question of dental jurisprudence, or the legal enactments governing or regulating the practice of dental surgery in the United States.

*First*, I will say that any enactment placed upon the statute books of our common country, or any of the independent States of our common country, should rest upon the immutable principle of equal justice to all and exclusive privileges to none.

*Secondly*, All laws that are intended to control the conduct of an individual or individuals in regard to any definite and universally uniform question in all of the States, should be absolutely the same in all particulars.

*Thirdly*, All such laws should be so plain and simple in their construction that the humblest citizen could comprehend them, and if necessary, administer the same in case he should be called upon so to do by the suffrage of the people.

*Fourthly*, The penal feature should be so well marked and imperative that none would dare disregard, only at their peril. Upon this feature, more than any other perhaps, depends the efficiency of all laws.

*Fifthly*, Each State should have the same enactments controlling the matter of dental education, making the requirements for graduation uniform in every particular, thus holding dental colleges up to a higher and better standard.

*Sixthly*, The first registration of an individual to practise should be made within twelve months after receiving a diploma from a regular dental institution of learning. The second or subsequent registrations in any other State of the Union should depend upon the presentation of a certificate from the State Board of Dental Examiners, that the individual held a diploma from a reputable dental college, and that he had engaged only in the reputable practice of dental surgery while remaining a citizen of the State in which he first registered.

Finally, the above synopsis, with other minor details, such as the regulation of fees and penalties, and the designation of proper tribunals to have jurisdiction over the questions involved, should make up the sum total of all that is embraced in the subject of dental legislation, in this or any other country.

In order that this may be accomplished, I would respectfully suggest that this Association appoint a committee of one member from each State, and that the National Association of Dental Examiners be requested to appoint one member from each State; also the Dental College Faculty Association be requested to appoint one member from each State having a dental college or colleges, to be known as a general legislative committee, whose duty it shall be to formulate and draft such a uniform law as could not fail to be satisfactory to all the States. That would result in the accomplishment of the most good, not only to the profession but to the public at large. That such committee be requested, through its representatives in the various States, to have this uniform law presented as nearly simultaneously in all the States as possible, and that this national organization, by resolution, unanimously request the hearty co-operation and support of the entire profession in the Union.

This work is of so much magnitude and fraught with so much interest to all departments of dental science and dental institutions in this country that it cannot be accomplished within a short period of time. Hence this legislative committee should be selected with great care, in order that there may be as little friction as possible, since the cavilling and contention by the profession over features of minor importance has been the greatest obstacle in the way of obtaining proper legislation for the practice of dentistry in this country.

This uniformity of laws is one of the reasons that could be offered at this time for creating out of the two organizations that are now in existence—to wit, the American Dental Association and the Southern Dental Association—one national organization, to be known by such name as would be appropriate and proper.

I would call your attention to this additional fact,—in order to stimulate discussion and action upon this important matter,—that there are many good and reputable citizens now engaged in the practice of dental surgery—and general medicine, so far as that is concerned—in some States of the Union who are deprived of citizenship in their States by virtue of the improper construction and requirements of the laws of certain States of this Union, virtually disfranchising them to a certain extent.

In view of the interest manifested in the matter of uniting the American Dental Association and the Southern Dental Association,

I would respectfully advise that our next meeting be held at Old Point Comfort, at the usual time, as the Southern Dental Association has adjourned to meet at that time and place, and they have expressed the thought that it would be fully agreeable to have our Association do likewise, in order that a full and free discussion of the question of consolidation be finally and permanently settled. Further, I would suggest that in case the consolidation cannot be accomplished, that this Association hereafter hold its annual meetings in the three grand divisions of our country,—to wit, the Northeast to have one meeting, the Northwest to have one meeting, and the South to have one meeting, thus alternating every year in order that the entire country may have the benefit resulting from this Association.

Since our last meeting, we have lost by death the following members: Dr. W. H. Dwinelle, of New York; Dr. P. G. Hunt, of Indiana; Dr. C. W. Spaulding, of Missouri.

I would respectfully call special attention to the reports of your committee on the work of the National Museum, in connection with the Army Medical Museum at Washington. The progress of the work of that committee seems to be satisfactory, and all that could be desired. As to their recommendation for an appropriation, I respectfully refer the whole matter to you as a body, and I would further suggest that the full report of this committee be published in our annual proceedings, in order that as much information may be disseminated upon this subject as possible.

The president's address was received and placed in the hands of the Publication Committee.

*Dr. Morgan.*—I move that it be referred to a suitable committee to take into consideration the suggestions therein contained.

The chair appointed Drs. Morgan, Brackett, and Fuller, as such committee.

Dr. Crawford then resumed the chair.

On behalf of the Committee on Credentials, Dr. Jackson read the list of delegates who have presented certificates.

Report received.

*Dr. Peirce.*—I would like to give notice that any one having a voluntary essay will please hand it in at an early date.

*Dr. Horton.*—I would like to make an announcement, if it is in order: Five years ago I announced that my son had perfected a process by which sensitive dentine could be excavated without pain. It was received at that time with an expression of doubt, but a promise was made that as soon as an apparatus could be made that would be safe to put into the hands of the members of this Association, I would bring it up before this society again. I would say now that we have obtained an operating-room at Dr. Rich's office in the Arcade, where any gentleman can call and examine this apparatus and test it, and later on I may ask an expression of opinion in regard to it.

*Dr. Bogue.*—I have had sent to me this morning a remarkable bit of prosthesis. I am called back to New York, and if it be the pleasure of this society to have me show it now, I will be glad to do so.

One of our members, Dr. Michaels, of Paris, several years ago made an apparatus to replace an amputated portion of the lower jaw. It was worn under the periosteum for about a year, and the patient then died, but the result was so remarkable that when next a patient presented himself to Dr. Payon, a prominent surgeon of Paris, requiring the amputation of portion of the bone, Payon went to Michaels to see what could be done. Payon put that attachment which I show you here into the man's shoulder. It has a rotary motion. After two years of wearing, Dr. George Allan and myself went to see this man. That was the model which was shown to Payon. From that another was made exactly like it, except that the wires were made of platinum instead of German silver.

*The President.*—Is the clinical history of this case published?

*Dr. Bogue.*—Yes; it has been published in Paris, and here is an account of it. A large photograph was made of the man, who is now alive and well.

Adjournment until 7.30 o'clock this evening.

(To be continued.)

